# Permalloy nanowires/graphene oxide composite with enhanced conductive properties

**DOI:** 10.1038/s41598-020-70512-1

**Published:** 2020-08-13

**Authors:** Diana M. Arciniegas Jaimes, Paulina Márquez, Alexandra Ovalle, Juan Escrig, Omar Linarez Pérez, Noelia Bajales

**Affiliations:** 1 CONICET, IFEG, Av. Medina Allende s/n, 5000 Córdoba, Argentina; 2 Department of Physics, Universidad de Santiago de Chile, 9170124 Santiago, Chile; 3 Center for the Development of Nanoscience and Nanotechnology, 9170124 Santiago, Chile; 4 Departamento de Fisicoquímica, Facultad de Ciencias Químicas, Universidad Nacional de Córdoba, Haya de la Torre esq. Medina Allende, X5000HUA Córdoba, Argentina; 5 CONICET, INFIQC, Haya de la Torre esq. Medina Allende, X5000HUA Córdoba, Argentina; 6 FAMAF, Universidad Nacional de Córdoba, Medina Allende s/n, Ciudad Universitaria, 5000 Córdoba, Argentina

**Keywords:** Materials science, Materials for devices, Sensors and biosensors, Atomic force microscopy, Scanning electron microscopy, Characterization and analytical techniques

## Abstract

Carbon–metal-based composites arise as advanced materials in the frontiers with nanotechnology, since the properties inherent to each component are multiplexed into a new material with potential applications. In this work, a novel composite consisting of randomly oriented permalloy nanowires (Py NWs) intercalated among the sheets of multi-layered graphene oxide (GO) was performed. Py NWs were synthesized by electrodeposition inside mesoporous alumina templates, while GO sheets were separated by means of sonication. Sequential deposition steps of Py NWs and GO flakes allowed to reach a reproducible and stable graphene oxide-based magnetic assembly. Microscopic and spectroscopic results indicate that Py NWs are anchored on the surface as well as around the edges of the multi-layered GO, promoted by the presence of chemical groups, while magnetic characterization affords additional support to our hypothesis regarding the parallel orientation of the Py NWs with respect to the GO film, and also hints the parallel stacking of GO sheets with respect to the substrate. The most striking result remains on the electrochemical performance achieved by the composite that evidences an enhanced conductive behaviour compared to a standard electrode. Such effect provides an approach to the development of permalloy nanowires/graphene oxide-based electrodes as attractive candidates for molecular sensing devices.

## Introduction

Composite materials arise as versatile candidates that could be designed to satisfy diverse technological applications due to their multifunctional behaviour. Since composites are obtained by combining two or more materials that have different attributes, such assembly provides to the composite of unique properties. In fact, several examples exist that have demonstrated the industrial development behind their production, being the concrete a well-known composite, where its strength under compression is improved by adding metal rods, wires, mesh or cables in order to create reinforced concrete. Most of the current composites have been made by carbon allotropes^1, 2^. In particular, when graphene is combined with the metallic copper, a new material around 500 times stronger than copper is achieved^3^. Likewise, composites constituted by graphene and nickel yield a strength larger than 180 times of nickel^4^. A soft Ni-rich ferromagnet^5, 6^, that is widely employed as a magnetic core material in diverse technological applications, such as magnetic recording heads^7–9^, microinductors and magnetoresistive random access memories (MRAM)^10, 11^, is the so called permalloy (Py), an alloy with about 20% iron and 80% nickel content. On the one hand, bulk Py exhibits large magnetic permeability, enhanced sensitivity in sensing devices^12^, as well as low coercivity and remanence, of interest in magnetic shielding^13^. In addition, it is a material with nearly zero magnetostriction, an attribute that promotes an easier integration in different devices^13^. Besides, the high Ni content provides an excellent corrosion resistance to the alloy. On the other hand, low dimensional Py structures, such as nanowires (NWs), can afford novel properties compared to 3-D atomic arrangements. As well, one-dimensional ferromagnetic wires display small size effects and a large shape anisotropy^14, 15^. Therefore, the strategy of decorating a carbonaceous matrix with such nanostructures that reveal a strong dependence on geometrical parameters, like diameter and length^16, 17^, supplies a key tool to design, tune and control the physical properties of composite materials for high-impact technology^18–20^, with ever-widen utilities, in order to obtain enhanced multiplexed attributes.

Being graphene an expensive carbon-based material to be composited, alternative low-cost sources that provide easy access and non-toxic nature arises, being the graphene oxide (GO), the oxidized form of graphene^21, 22^, one of the most currently investigated. This chemically modified graphene contains oxygen functional groups such as epoxides, alcohols, and carboxylic acids, joined with structural defects^23^. The large number of epoxide and OH functional groups, make the GO hydrophilic and dispersible in aqueous media with long-lasting stability^24^. Moreover, GO behaves as insulator or semiconductor, depending on the oxidation degree. Therefore, GO is considered as an interesting matrix to create composites for multiple applications, ranging from biomedicine due to its antibacterial response^25^, environmental remediation because it allows removing the arsenic in water^26^, energy storage in supercapacitors as electrode^27, 28^, metal catalysis as support^29^, to the manufacture of screens^30^, and other flexible devices^31^. Motived by these multiple functionalities, intensive research has been tackled in the last years to develop GO-based composites^32, 33^, being its use as molecular sensors a trending driven force, since these new materials could afford stability, selectivity^34, 35^, as well as large surface area that provide anchorage points due to the presence of functional groups on the surface, that further impact on the simple scalability to develop real devices^36–38^.

In this work, Py NWs/GO composites were obtained by decorating multilayered GO thick films with ~ 30 nm in diameter and 1 µm long Py NWs, intercalated with random distribution among the GO layers. Py NWs were synthetized by electrodeposition inside anodic aluminium oxide (AAO) templates, while the carbonaceous matrix was prepared using commercial GO after several cycles of sonication and sequential deposition steps. Morphological, structural, magnetic, chemical composition and electrochemical characterizations of the individual components as well as the whole composite were performed. This integral study reveals an innovative electrochemical response obtained from the Py NWs/GO composite that discloses its potential application as molecular sensor for multiple uses.

## Results and discussion

### Morphological and structural characterization

**Figure 1:**
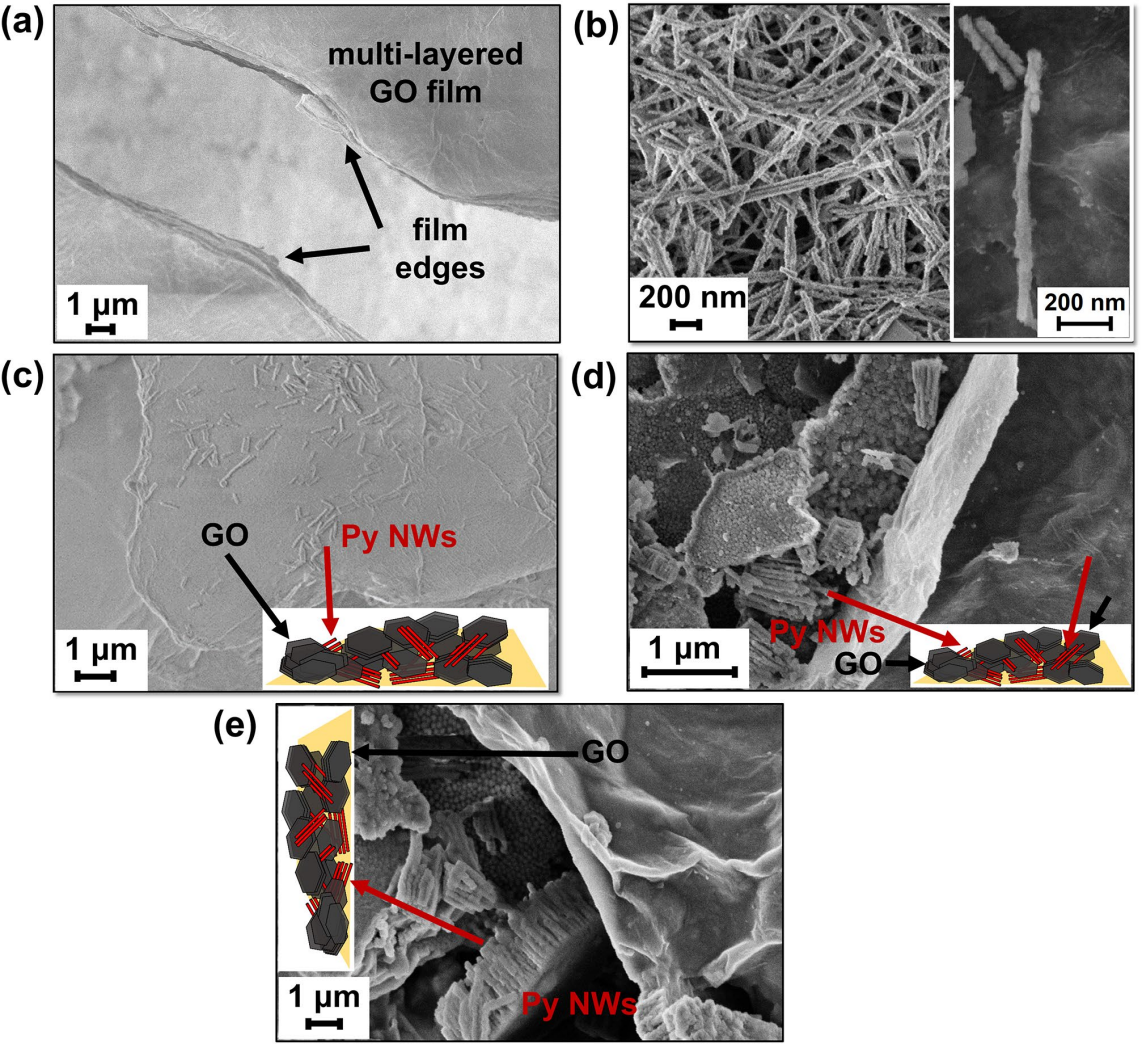
SEM images of (**a**) GO thick films, (**b**) decoupled Py NWs, and (**c**), (**d**) and (**e**) different views of the Py NWs/GO composites, including schemes as guides to the eye for an easier recognition of each composite component. Inset in (**b**): representative isolated Py NWs decoupled from AAO template.

Figure 1 displays the micrographies obtained by means of scanning electron microscopy (SEM) of multi-layered GO thick films (Fig. 1a), decoupled Py NWs (Fig. 1b) and Py NWs/GO composites (Fig. 1c–e). On the one hand, Fig. 1a discloses two well-separated GO thick films that evidence broad lateral dimensions of several micrometers of extension and edges of different widths, being these latter discernible through the projected side views of both multi-layered films. Such large films are obtained from the random stacking of assorted individual GO flakes that operate as building blocks, which average lateral size is  ~ 1 µm, as it is shown in Fig. S1 (see Supporting Information). On the other hand, decoupled Py NWs are shown in Fig. 1b. A high density of isolated Py nanowires is achievable by dissolving the alumina template. Nonetheless, it is worth noting that the decoupling procedure was conducted after an exhaustive structural characterization performed on several samples in order to determine the appropriate alumina-dissolution time that leads to a considerable number of nanowires, but preserving the morphology and magnetic nature of the Py nanostructures. Thus, the inset in Fig. 1b shows the SEM image of an individual decoupled Py NW obtained after the removal of the AAO membrane. In this latter, a representative Py NW of ~ 1 µm in length and ~ 30 nm in diameter with serrated morphology can be observed. Besides, Py NWs of shorter lengths are also present since they were shattered during the decoupling process.

SEM images of the resultant composite obtained by the assembly between the randomly orientated Py NWs and the multi-layered GO matrix are shown in Fig. 1c–e. While Fig. 1c displays that the GO film surface is decorated with several decoupled Py nanowires arrays, most of them oriented parallel to the GO surface plane and probably overlapped among some GO layers, Fig. 1d,e reveal the presence of alumina-embedded Py NWs even after the decoupling process. In fact, since the nanowires morphology and magnetic behaviour should be mainly preserved during the chemical treatment used to separate the Py NWs from the AAO template, and at the same time, a large amount of decoupled Py nanowires is desired, residual alumina-embedded nanowires are observed as well. SEM images evidence that such Py agglomerates are located around the edges of the multi-layered GO thick films and also intercalated among their layers. This is probably due to the anchorage of Py NWs to GO layers promoted by the functional groups in GO. Schemes of the formed composite were included to the Fig. 1c–e as a guide to the eye for an easier recognition of each component in the SEM images.

**Figure 2:**
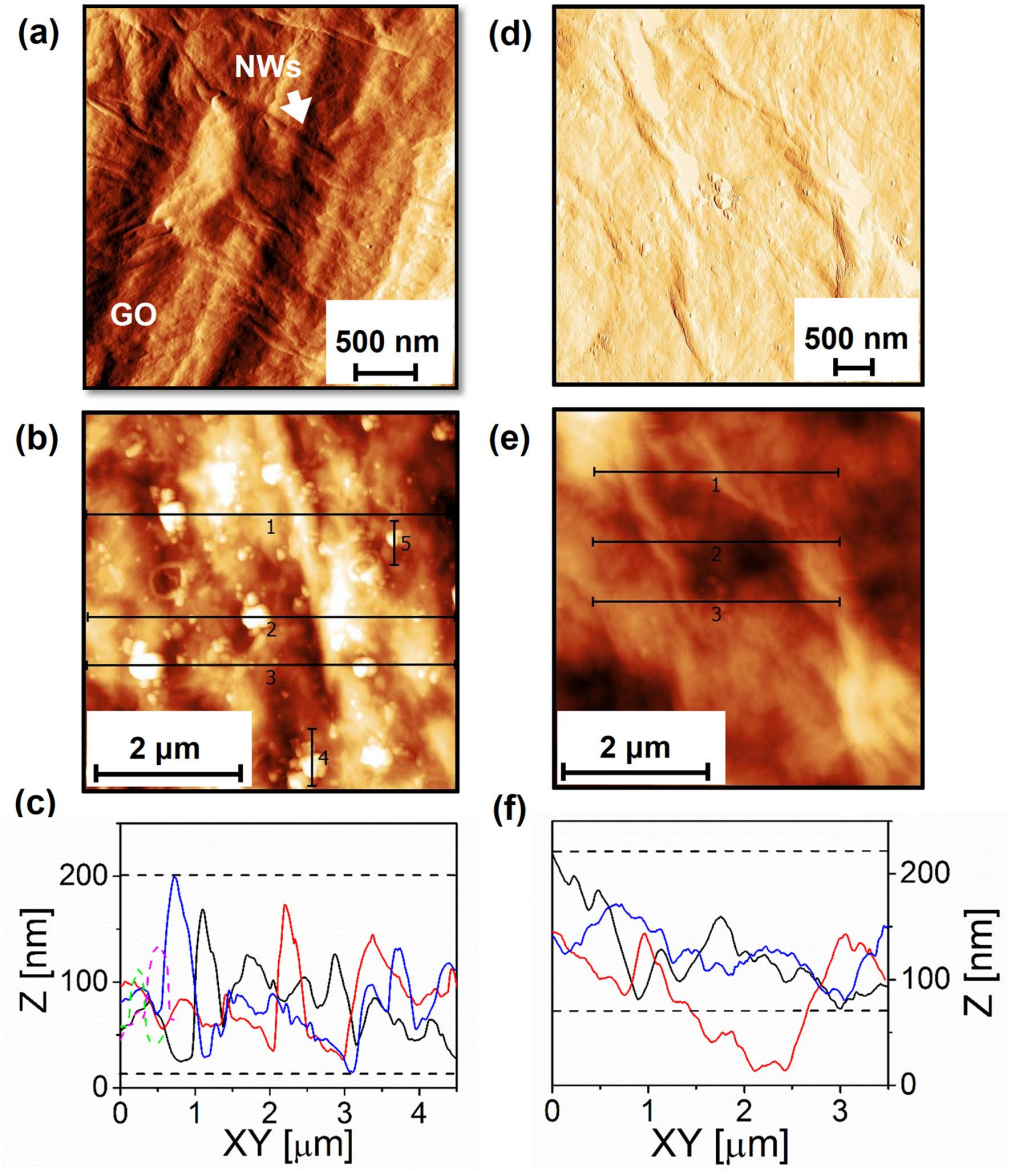
For composite: (**a**) AFM image of amplitude, (**b**) AFM topographic image and (**c**) its corresponding AFM height profile. For GO: (**d**) Amplitude image, (**e**) AFM topographic image and (**f**) its corresponding AFM height profile. For (**c**) and (**f**): Continuous lines correspond to Profile 1 (black), Profile 2 (red), Profile 3 (blue); and Dashed lines in (**c**): Profile 4 (Magenta), Profile 5 (Green).

Amplitude images of the composite and the GO films, obtained by means of atomic force microscopy (AFM), are shown in Fig. 2a,d, respectively. Figure 2a reveals the morphology of several unidimensional structures of around 1 µm long, which are consistent with the expected one for the Py nanowires. Most of these nanostructures seems to be lying on the GO surface with certain grade of intercalation among flakes, suggesting that are mainly oriented parallel to the GO supporting plane, in good agreement with our hypothesis discussed above for SEM images. In comparison with this latter, Fig. 2d discloses a wavy morphology for the surface of the GO thick films free of nanowires, effect that is attributed to the overlapping of GO flakes that are stacked in random orientation due to the preparation method of sequential drops. On the other hand, AFM topography images of the composite and GO films are shown in Fig. 2b,e, respectively, while their corresponding height profiles are depicted in Fig. 2c,f. The topography of the composite evidences the presence of an aleatory distribution of tridimensional nanoagglomerates with different sizes that are pinned to the GO wavy surface strong enough to survive several scannings, in tapping mode, without being displaced from their initial positions. A thoughtful statistical data treatment performed on several topography images of the composite arises an average height profile of ~ 180 nm in the scanned areas. It is important to remark that even when the mean height for the composite appears to be dominated by the rippled GO film height, the nanoagglomerate heights can be resolved from the height profile depicted in Fig. 2c, which provides evidence for small heights of around 30 nm to larger ones of almost 100 nm, being the lateral extension of hundred nanometers. These values are in good agreement for isolated Py NWs, clusters of Py NWs as well as embedded-alumina Py NWs when they are oriented in a parallel configuration with respect to the GO surface. Broken Py NWs of shorter lengths than 1 µm could also contribute. Such accuracy in the determination of the NWs parameters is not achievable in the SEM images of the composite. Likewise, the average height profile for the GO matrix (Fig. 2f) free of nanowires was ~ 150 nm in the scanned areas. Here, it is important to note that heights larger than 2 µm are not achievable by the used AFM scanner. In such cases, we have changed the scanned region, to perform the measurements of both samples in the same conditions in order to establish a comparative analysis. This striking difference that arises from the comparison between the average heights obtained for the composites and GO films, measured in the same scanning conditions, provides complementary support to the hypothesis that the assorted distribution of magnetic nanowires (single or in clusters) are mainly anchored parallel to the wavy surface of the GO sheets. Additional evidence to asses this latter statement is supplied by our magnetic measurements (see below).

**Figure 3:**
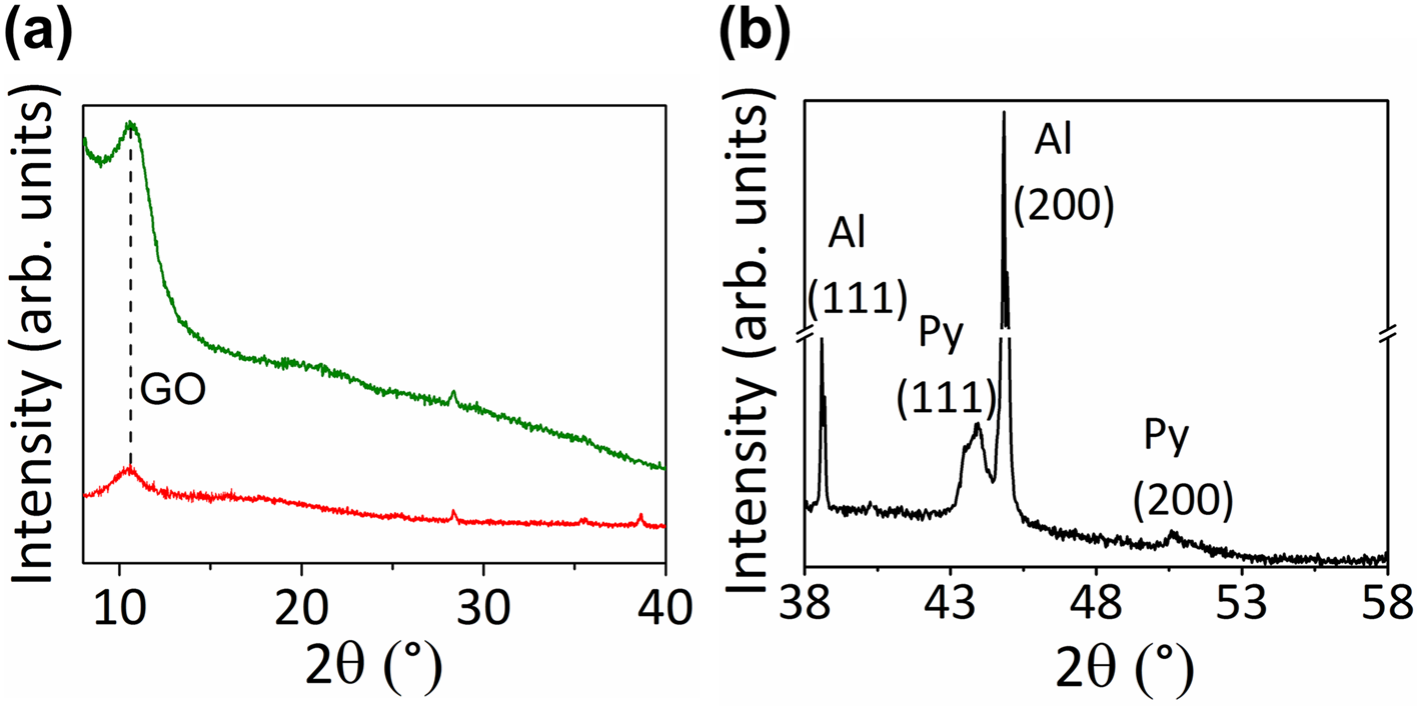
XRD pattern data of (**a**) GO (green) and composite (red) samples and (**b**) Py NWs inside AAO template.

The X-ray diffraction patterns (XRD) of GO and of the composite are shown in Fig. 3a, while in Fig. 3b the XRD pattern of Py NWs inside the AAO template is depicted. In the XRD patterns of GO and composite displayed in Fig. 3a, the presence of well-resolved broad peaks located at around 2θ = 10° is assigned to GO thick films in good agreement with previous reports^34^. The peaks of very low intensity centered at ~ 2θ = 28°, discernible in both samples, are attributed to Si(111), which is the material of the sample holder. On the other hand, since no signal expected for Py NWs^13, 39^ is observed in the XRD pattern of the composite, probably due to the signal intensity coming from the low density of intercalated nanowires is under the limit detection of the technique, we have performed XRD measurements in the AAO template containing a huge amount of Py NWs in order to analyze the phase in which the nanowires crystallize (Fig. 3b). In this latter, wide peaks of low intensities can be observed at approximately 43.7° and 50.7° in 2θ, which corresponds to permalloy (111) and (200), respectively. The large peak around 44.9° denote the presence of residual aluminium corresponding to remaining crystals that survive to the Al substrate dissolution. Permalloy peaks are detected, even though their volume is smaller than that corresponding to the residual Al substrate.

**Figure 4:**
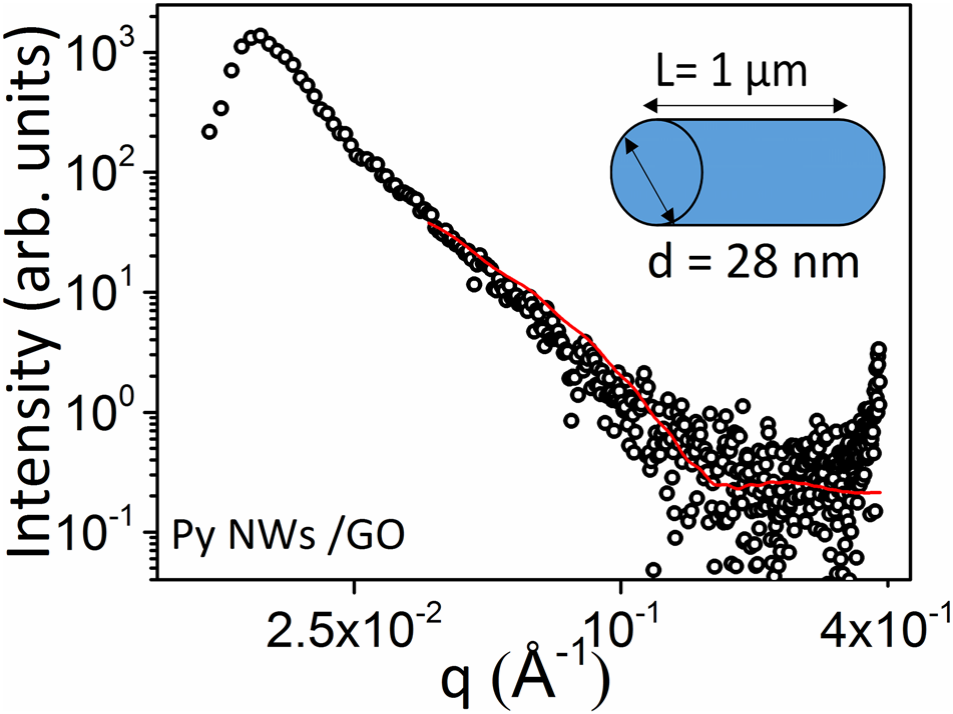
Experimental (black circles) and fitted (red line) SAXS data of composite assuming a cylindrical shape.

Small-angle X-ray scattering (SAXS) measurements were performed on the composite sample in order to provide additional evidence for the distribution of particle sizes in the composite. Thus, Fig. 4 displays the SAXS intensities, I(q), where q = 4π (sinθ)/λ, versus the modulus of scattering vector, q, in the logarithmic scale for Py NWs/GO composite. From the fitting of the SAXS experimental curve, an estimated size of the nanostructures assuming the presence of different geometries was performed. Using a cylinder as a shape-model fitting (see Equation 1 in the Supporting Information) and keeping the length of the cylinder fixed as L = 1 μm, an excellent agreement was obtained for the nanowire shape with an adjusted diameter value of d = 28 nm, which is a pretty result fully consistent with the average diameter obtained from SEM measurements for the Py nanowires (Fig. 1b). In addition to this latter good agreement achieved for the average value of the NW diameter, Fig. 4 also hints the large dispersion of the data set with regard to the diameter variation of the NWs as well as their random orientation.

### Spectroscopic characterization

**Figure 5:**
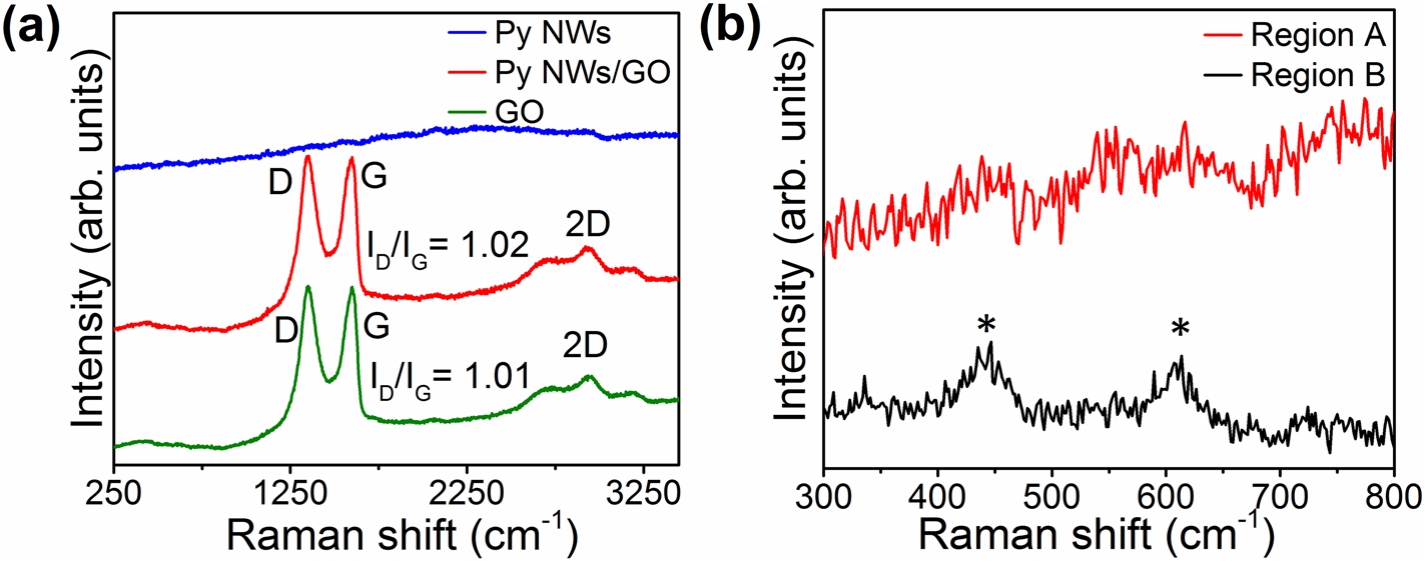
(**a**) Raman spectra of GO (green), Py NWs/GO composite (red), and Py NWs (blue). (**b**) Raman spectra of two different regions (A: red, B: black) in the decoupled Py NWs sample.

Figure 5a shows Raman spectra performed on the as-prepared substrate/Py NWs and substrate/Py NWs/GO composite samples, which have been acquired from 250 to 3250 cm^−1^, while Fig. 5b displays Raman spectra for two different regions in the Py NWs sample, which have been acquired in a shorter range with the aim of exploring whether the expected bands for the permalloy can be resolved.

On the one hand, a comparative analysis of Raman spectra of the GO matrix with those of the Py NWs/GO composite indicates that the chemical nature of GO in the composite is preserved, since the relative intensities between that of the typical D band (I_D_) at ~ 1349 cm^−1^ and the one of the G band at ~ 1587 cm^−1^ (I_G_) are quite similar and reaches a value of I_D_/I_G_ ~ 1 for both samples. Likewise, the 2D band at ~ 2680 cm^−1^ is present in both Raman spectra^40–42^. On the other hand, the bands expected for the Py NWs are not resolved in the corresponding Raman spectrum (Fig. 5a, blue line) when it is acquired from 250 to 3250 cm^−1^. This is probably due to the low density of decoupled nanowires in the Py sample^40^. Therefore, in order to verify the appearance of the Ni and Fe bands on the Py NWs sample, several Raman measurements were performed in two well-separated different regions that have been called *Region A* and *Region B*, respectively. Keeping in mind that the laser spot is ~ 1 µm in diameter, a statistical treatment applied to both regions leads to the identification of small bands (marked with asterisks in Fig. 5b) in the 70% of the measurements (Region B in Fig. 5b), while in the 30% of the cases, no bands corresponding to Ni and Fe were found (Region A in Fig. 5b). These bands were positioned in approximately 445 and 613 cm^−1^, and have been associated to the Ni–O and Fe–O, respectively^43, 44^. This result suggests a certain degree of oxidation of the NWs in the sample as it was previously reported^43, 44^.

**Figure 6:**
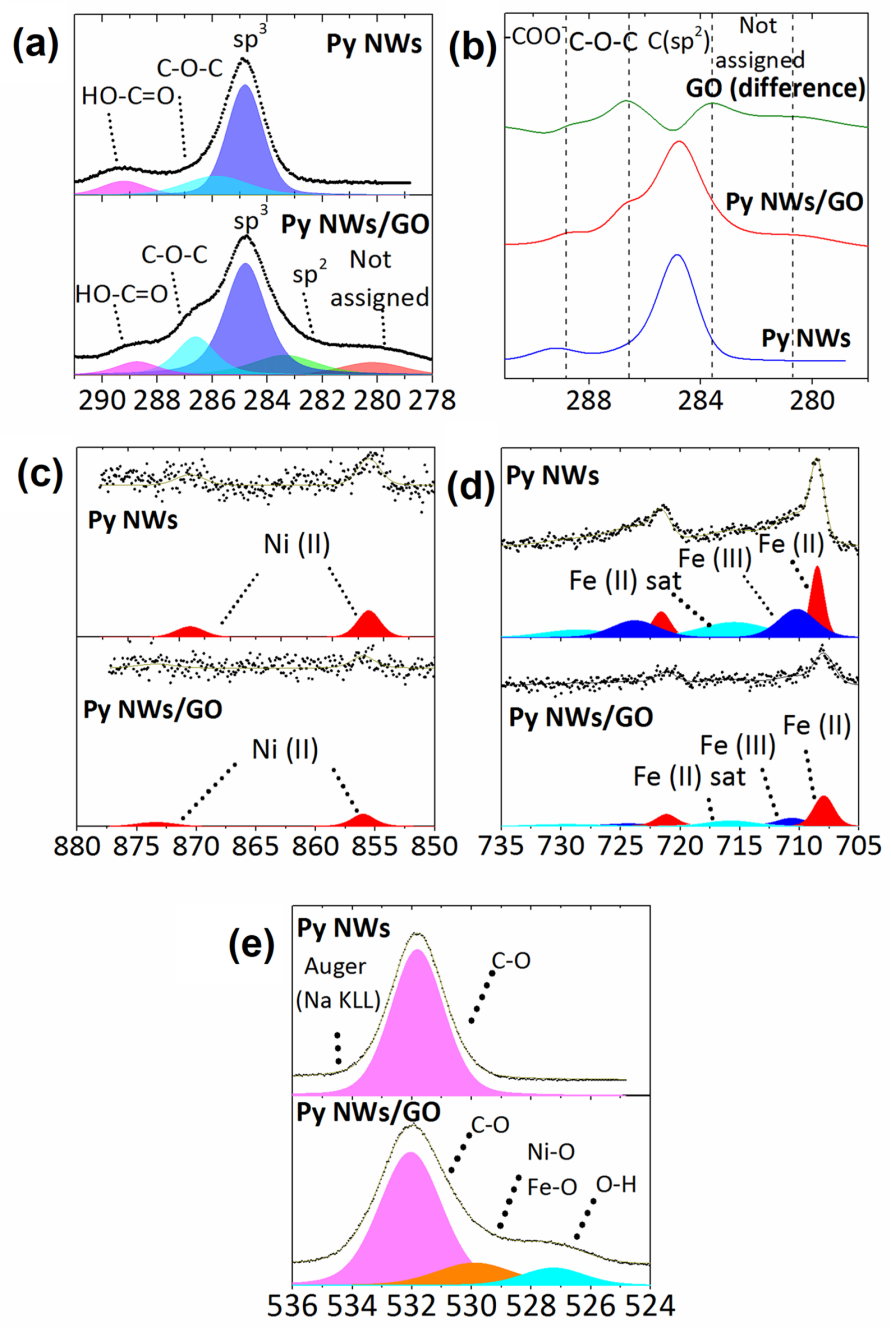
XPS spectra (Intensity in arb. units vs. Binding energy in eV) of Py NWs and Py NWs/GO for (**a**) C1s, (**b**) difference of C1s, (**c**) Ni2p, (**d**) Fe2p and (**e**) O1s.

X-ray photoelectron spectroscopy (XPS) measurements gives information about the surface chemical composition of the Py NWs/GO composite and Py NWs samples. In the XPS spectra displayed in Fig. 6a four characteristic C peaks of GO^45, 46^ appear at binding energies of 288.7, 286.6, 284.8, 283.4 eV for Py NWs/GO composite, which corresponded to carboxyl/hydroxyl, epoxy groups, C sp^3^ and C sp^2^, respectively. A not assigned peak appear at 280.2 eV, that could be associated to a possible anchorage between GO and NWs (C-metal)^47^ or due states that are pπ-like (C2p–O2p) bonding character from O lone-pairs^48^. The presence of some carbon species in Py NWs is probably due to sample manipulation and possible sample or chamber contamination, which can be seen in the difference of the spectrum of C in Fig. 6b, showing the main contribution of GO in the composite. Figure 6c, d shows the spectrum of Ni and Fe, respectively. A doublet was observed for Ni2p in ~ 856.0 eV and ~ 873 eV^49^; and a main doublet at ~ 708.0 and ~ 721.5 eV and another one with lower intensity at ~ 710.2 eV and ~ 724.1, assigned to Fe(II) and Fe(III) species, respectively^49^. Due to both, the low signal in the spectra of Ni and Fe and the electron free path depth (< 10 nm), the assignation of peak contributions was not easy but it was concluded that Ni and Fe were probably partially oxidized at the Py NWs surface (NiO, Ni(OH)_2_ and Fe_x_O_y_ species). This is probably due to the synthesis or exposure to ambient air although XRD data have not shown signature of Ni–Fe oxide phase formation due to the low quantity in the sample^43^. In the O1s spectra from Fig. 6e could be seen for Py NWs/GO composite, the O1s binding energy has three components at 532, 529.9 and 527.3 eV that can be assigned to C–O, Ni–O or Fe–O^50, 51^ and O–H^52^, respectively. Meanwhile, for the Py NWs only one peak at 531.9 eV for a typical C–O bond due to a possible contamination of the sample was found. Table S1 in Supplementary Information summarizes all of the data relative to the fitting curves of each spectrum, such as Binding Energy (B.E.), Full Width at Half Maximum (FWHM), Lorentzian and Gaussian mix percentage (L/G Mix) and the total area ratio (Area). The O–H signal could be assigned to the hydroxyl groups presence in GO or Py NWs surface, or due to the possible hydrogen bridge between them, indicating the anchoring nature.

### Magnetic characterization

**Figure 7:**
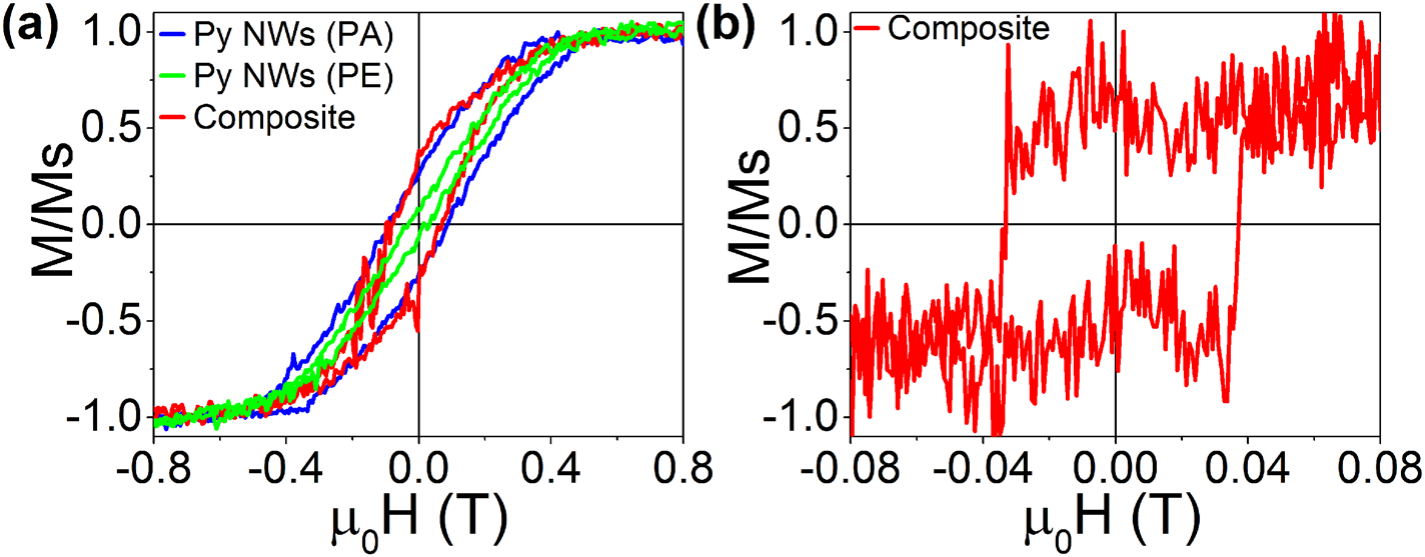
Room temperature hysteresis loops of composite with measurements of (**a**) VSM and (**b**) MOKE. For (**a**) measurements are performed with the applied field parallel (PA) to the NWs long axis inside AAO (blue line), perpendicular (PE) to the NWs long axis inside AAO (green line) and parallel to the substrate of composite (red line). For (**b**) measurements are realized parallel to the substrate of the composite (red line).

Magnetic measurements were performed by means of a vibrating sample magnetometer (VSM) with the purpose of comparing the magnetic properties of the composite with that exhibited by the ordered Py NWs inside the AAO alumina membrane. Thus, the room-temperature hysteresis loops of Py NWs/GO composite and the Py NWs embedded in the AAO template where depicted in Fig. 7a. The magnetic field was applied parallel (PA) and perpendicular (PE) to the long axis of the Py NWs since their orientation is well-defined due to the large ordering range provided by the alumina template, used as supporting substrate for this case. For the composite, the magnetic field was applied parallel to the substrate because to the low density of the Py NWs that are randomly oriented. In fact, PA and PE configurations can not be defined for the composite since it is not feasible to establish how the long axis of the NWs are oriented respect to the Au/Al substrate. Regarding the substrates, paramagnetic (Al) and diamagnetic (Au) contributions were removed in all cases.

From Fig. 7a it can be seen that the composite has similar values of coercive field, Hc, ~ 0.09 T, and remanence magnetization, Mr/Ms, ~ 0.30, compared to those exhibited by the Py NWs with the easy magnetization axis parallel (PA) to the NWs long axis. In fact, PA configuration reaches a higher Hc value than the one obtained for the PE orientation in the Py NWs sample. On the one hand, this result reveals that, despite of very low density of decoupled Py nanowires in the composite, its magnetic properties mirror the magnetic behaviour disclosed by a larger density of well-ordered Py NWs when their easy axis is parallel to the applied field. On the other hand, these results not only afford additional support to our hypothesis regarding the parallel orientation of Py NWs respect to the GO film, where they are anchored in the composite (Figs. 1c–e, 2a,c), but also hint that the stacking of GO sheets tends to assembly in average parallel to the substrate plane.

In addition, the surface magnetic behaviour of the Py NWs/GO composite has been investigated by longitudinal magneto-optical Kerr effect (MOKE) magnetometry. It is worth to note that to achieve this goal meant a meticulous search due to the random distribution of low density of magnetic nanowires on the composite surface. As a result, the characteristic hysteresis loop of a few isolated Py NWs was achieved when the external magnetic field was applied parallel to its axis as it is shown in Fig. 7b. The high noise in the signal is due to the fact that the environmental vibrations easily defocused the center of the laser spot on the surface of the nanostructure. Besides, the area illuminated by the laser is larger than the diameter of the nanowires.

A comparison between Fig. 7a,b clearly unveils that the Hc value obtained for the composite by means of MOKE is lower than that measured by VSM, while the opposite trend is observed for the squareness of the hysteresis loop. In the first case, this difference is probably due to the larger size of the laser spot (~ 2 μm) respect to the NW length and diameter. Nonetheless, it is important to note that, even when there were areas where nothing could be measured, the contribution of a few Py NWs was successfully detected. Regarding the squareness, the high dispersion of the NWs on the GO surface is resembled by a more pronounced squareness obtained with MOKE, due to the non-interaction among the well-separated NWs, in comparison with that observed for the composite measured by VSM.

### Electrochemical characterization

To study the electrical behaviour of the samples, Electrochemical Impedance Spectroscopy (EIS) measurements using ferro/ferricyanide species as redox probe, were performed. Because the ferro/ferricyanide electron transfer reaction $\left[ {{\text{Fe}}\left( {{\text{CN}}} \right)_{6} } \right]^{4 - } \left( {{\text{aq}}} \right) \to \left[ {{\text{Fe}}\left( {{\text{CN}}} \right)_{6} } \right]^{3 - } \left( {{\text{aq}}} \right) + ({\text{e}}^{ - } )$ has a well-know kinetic behaviour, changes in the electrochemical response may be attributed to modifications in the electrode conductive properties^53^.

**Figure 8:**
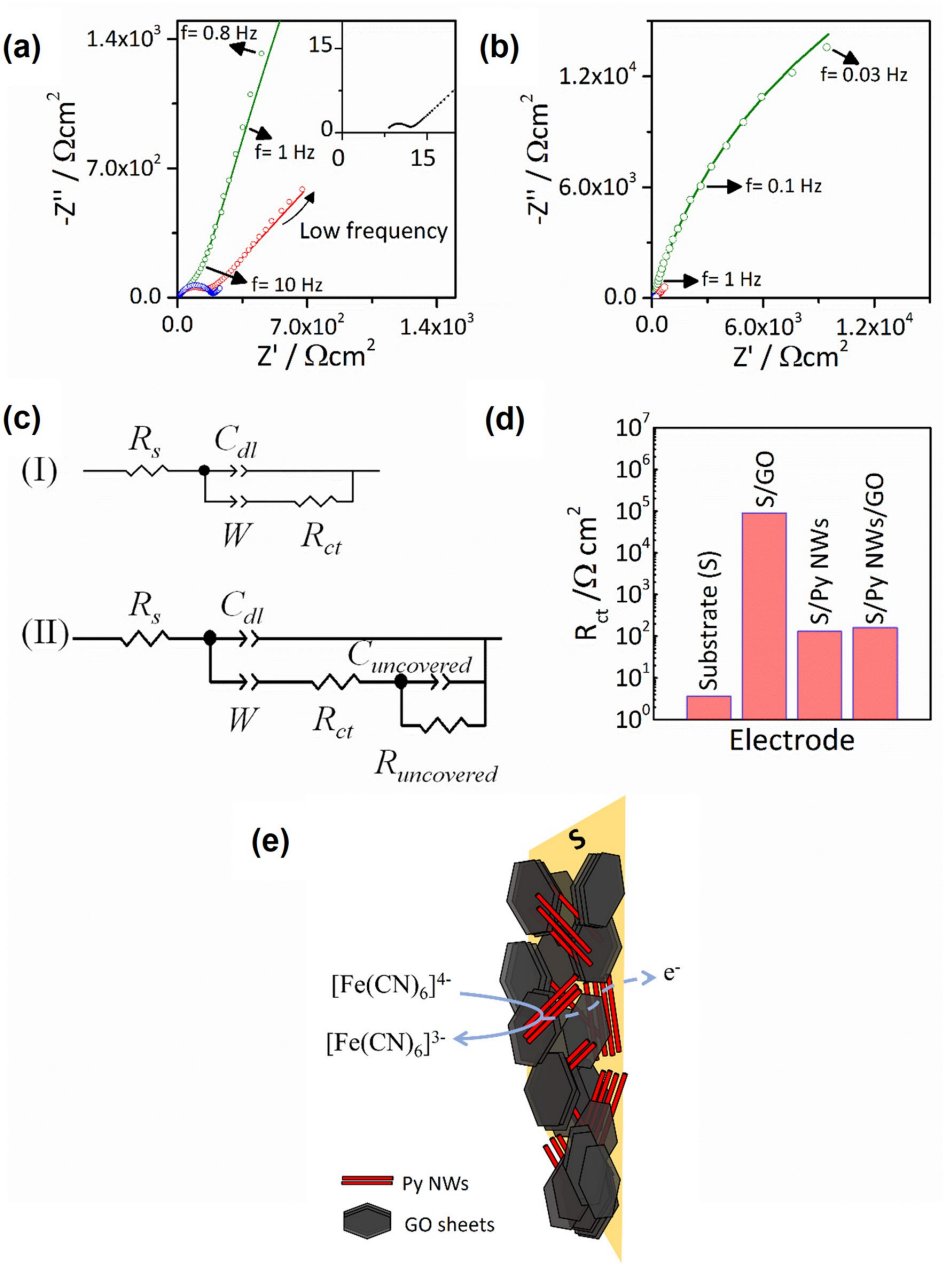
(**a**) and (**b**) Nyquist diagrams in 10 mM [Fe(CN)_6_]^3−^/[Fe(CN)_6_]^4−^ solution of experimental (open circles) and fitting data (lines) of GO (Green), Py NWs/GO (red) and Py NWs (blue). Inset in (**a**): Substrate (black). (**a**) and (**b**) show the same data in different frequency domain. (**c**) Circuits used for fitting. (**d**) Charge-transfer resistance of the different electrodes. (**e**) Proposed sketch.

Figure 8a,b show complex plane representations (Nyquist diagrams) at different scales of the impedance response for the substrate, substrate/GO, substrate/Py NWs and substrate/Py NWs/GO composite electrodes in 10 mM [Fe(CN)_6_]^4−^/[Fe(CN)_6_]^3−^ solutions. Except for substrate/GO sample (green data), at high frequencies the spectra in Fig. 8a,b show a well-defined semicircle followed by a straight line with 45° slope at lower frequencies. This impedance behaviour is the response expected for an electron transfer reaction controlled by the semi-infinite linear diffusion of species from the bulk electrolyte to the electrode surface. This response is usually analyzed in terms of the Randles equivalent electrical circuit (Fig. 8c-I), which takes in account the solution resistance (R_s_), electrochemical double layer capacitance (C_dl_), charge transfer resistance (R_ct_) and the Warburg diffusional element (W)^53^.

Lines in Fig. 8a,b are the best fits obtained using the Randles circuit except for substrate/GO sample, which impedance response shows a different behaviour at low frequencies. For this sample, impedance was fitted using the circuit shown in Fig. 8c-II which takes in account the electron charge transfer of the redox probe at uncovered areas of the electrode as an additional RC coupling. This behaviour does not occur on Py NWs nor in the composite modified electrodes because their conductivities would be greater than that of GO and a more homogeneous surface is present. Table S2 in the Supporting Information summarizes the values of the electrical elements obtained from the fittings for the set of electrodes shown in Fig. 8a,b.

In order to test the reproducibility of the impedance results, a set of ten samples for each electrodes was analyzed. Figure 8d shows the average R_ct_ values obtained. Despite of the surface inhomogeneities that can appear as a consequence of the preparation method or partial surface oxidation of the Py NWs, a defined R_ct_ trend is determined with the following mean and dispersion values: (3.6 ± 0.4) Ω cm^2^ for the substrate, (90 ± 10) * 10^3^ Ω cm^2^ for GO, (130 ± 30) Ω cm^2^ for Py NWs and (160 ± 7) Ω cm^2^ for the composite. It is noticiable that the charge transfer resistance for the GO coating is around four orders of magnitude higher than the one for the conductive substrate, in agreement with its isolating behaviour. Contrarily, an important improvement of the conductivity, in comparison to substrate/GO, is obtained for the substrate/Py NWs/GO composite electrodes related to the Py NWs presence.

Thus, our SEM and AFM results indicate that the Py nanowires are distributed both on the surface as wells as around the edges of the multilayered GO thick films. Also, magnetic and spectroscopic results indicate that Py NWs are preferentially observed among GO layers in a parallel arrangement due to the chemical interactions that take place between the chemical groups at the surface of each component. In addition, the modification in the electrochemical behaviour of the usually used redox probe (ferro/ferricyanide species) indicates that exist a random distribution of decoupled NWs as well as GO sheets on the substrate, which allows preferentially the electron conduction to the substrate mediated by the Py NWs. Therefore, microscopic, magnetic, spectroscopic, and electrochemical results allow us to imagine a physical representation of the composite electrodes, as it is shown schematically in the Fig. 8e.

The observed improvement in the conductive properties for the present Py NWs/GO composite using GO as matrix also hints that this carbon-based material could lead to the development of a cheaper alternative than the use of graphene^54, 55^ in molecular sensing nanostructured devices^56–59^.

To summarize, we have reported a low-cost method to obtain Py NWs/GO composites by self-assembly of Py nanowires and graphene oxide sheets. Structural and morphological results indicate that Py NWs are distributed both on the surface as wells as around the edges of the multi-layered GO thick films. Magnetic and spectroscopic results indicate that Py NWs are preferentially observed between GO layers in a parallel arrangement. Also, the anchorage between the composite components may be due to the chemical groups present both at the GO and the partially oxidized Py NWs surfaces. These results allowed to gain insight into the physical and chemical attributes of the composite as well as the individual components, providing relevant information to explore its electrochemical behaviour. In this sense, the modifications in the electrochemical behaviour of a usually employed redox probe also indicate that exist an almost homogeneous distribution of NWs and GO on the substrate, which allows preferentially the electron conduction to the substrate mediated by the Py NWs. Thus, Py NWs/GO-based electrodes arise as promising candidates in the development of cheap and stable molecular sensing nanostructured devices^54–59^.

## Methods

### Sample preparation

Anodic aluminium oxide (AAO) templates with 1 μm of length (see Fig. S2 in the Supporting Information) and pores of 20 ± 2 nm in diameter were obtained by the conventional two-step anodization process^35^, using high purity Alfa Aesar (99.997%) aluminium with 100 μm of thickness. As a previous step to the anodization, the aluminium foil was cleaned using acetone in ultrasonic bath and electropolished with HClO_4_:EtOH in 1:3 v/v^36^. The anodization process was carried out in a 0.3 M sulfuric acid solution at 2 °C by applying a DC voltage of 20 V during 20 min and 4 h in a first and second anodization step, respectively. After the first anodization step, the Al_2_O_3_ layer was dissolved for 10 min in a 0.2 M Cr_2_O_3_ and 0.4 M H_3_PO_4_ solution at 60 °C. Further drops of H_3_PO_4_ in 10% v/v in contact with the AAO surface during 15 min increased the average pore diameter to a final value of 28 ± 2 nm. This procedure promotes the reduction of the barrier layer thickness but preventing the collapse of the AAO template.

An electrochemical cell with two-electrodes was used for the synthesis of Py NWs by AC electrodeposition at room temperature. The bottom face of AAO template was coated with Au in order to use it as working electrode, since it helped to improve the electrical conductivity, and a graphite bar as a counter electrode. Peak to peak voltage amplitude of V_PP_ = 16 V and a f = 200 Hz during 1 min were applied. The electrodeposition was carried out in an aqueous electrolytic bath of 0.1 M NiSO_4_·6H_2_O, 5 mM FeSO_4_·7H_2_O and 0.2 M H_3_BO_3_ to improve conductivity.

The composite that we have aimed to investigate is formed by a multi-layered GO thick film, decorated with permalloy nanowires (Py NWs). For the composite preparation, the mechanical support was supplied by low cost commercial aluminium (paramagnetic material) foil (99%) of 100 µm thick, which has been coated with Au (diamagnetic material) of 100 nm thick to ensure the electrical conductivity. Such Al/Au plataform was used as a supporting substrate, so it is referred as to just “substrate (S)” along the present work. Thus, not only the composite but all the samples (GO films, permalloy nanowires and composites) were prepared on a robust and conductive substrate constituted by aluminium and gold to perform the several characterizations. Over this substrate, 10 µL of aqueous suspension for each compound was deposited over the support up to reach 80 µL for each one. For Py NWs/GO composite, 10 µL of GO diluted (0.04 mg/mL of commercial Graphenea) was dropped and when the drop was fairly dry, 10 µL of Py NWs was deposited. These NWs were previously decoupled from the AAO membrane using 1 M NaOH and dispersed by ultrasound to avoid agglomeration. This dripping procedure was repeated sequentially up to reach 80 µL of each compound. The non-active surface was covered with an insulating coating due to its non-electrical and non-magnetic signals, which are important features to take into account when performing the sample characterization. The scheme of the procedure to synthesize the Py NWs/GO composite can be seen in Supporting Information Fig. S3.

### Structural characterization

A Σigma Carl Zeiss field-emission scanning electron microscope (FE-SEM) was employed to perform the morphological characterization of each component and composite. Previously to the measurements, the samples were sputtered with 8 nm thick Cr coating in order to prevent the sample charging. The samples were irradiated with energies of 3 keV for SEM micrographies. A magnification of 30 k× and 300 × was used to obtain the micrographies of Py NWs and GO layers, respectively, and 8.5 k× and 15 k× for the composite.

Agilent Technologies 5500 atomic Force Microscope (AFM) was used to study the topography and amplitude of the formed composite. Intermittent mode images were acquired using Silicon probes (Tap300Al-G, with 30-nm aluminium reflex coating and a nominal tip radius of 10 nm) from Budget Sensors, with a nominal resonant frequency of 320 kHz. A 512 pixel × 512 pixel resolution and a 5 μm × 5 μm of scanning area were chosen. Gwyddion 2.50 software (https://gwyddion.net/) was used for image analysis.

Two X-ray diffractometers with Bragg–Brentano geometry and Cu Kα (λ = 1.5418 Å) radiation were used. A Philips PW1800/10, with standard working parameters of 40 kV and 30 mA, was used to analyse the phases present in the samples. The X-ray diffraction (XRD) pattern of the NWs inside the AAO template was measured using a PANanalitycal X'Pert Pro diffractometer (40 kV, 40 mA).

A Bruker N8 HORIZON Small Angle X-ray Scattering System (SAXS) with Cu Kα (λ = 1.5418 Å) source, 2D VÅNTEC-500 and MONTEL optics was used to study the shape of the nanostructures in the composite. The data were acquired in the q-range from 0.012 to 0.380 Å^−1^ with a measurement time of 2 h at room temperature in vacuum (2 mbar). The analysis and data processing were achieved using the Bruker DIFFRAC.SAXS program. To make the measurement, the composite was decoupled from the Au/Al substrate, because the SAXS measures in transmission mode.

### Spectroscopic measurements

Raman spectra were measured with a confocal microscope coupled with a Raman spectrophotometer (Horiba Jobin–Yvon LabRam HR) using a 100 X optical objective. The excitation wavelength, laser power and attenuator filter were 514.51 nm, 2.8 ± 0.2 μW, and 10%, respectively.

The X-ray Photoelectron Spectroscopy (XPS) of K Alpha Thermo Fisher Scientific has a monochromatic X-ray source (Al Kα = 1486.6 eV), capable of high resolution analysis. A spot size of 300 µm and an energy step of 0.1 eV was used. This technique was used to obtain the oxidation state of Py NWs and analyse the chemical environment and possible chemical interactions between different species. Before analysing the data, a shift of all spectra was performed, taking into account the position of the adventitious C (284.8 eV). The CasaXPS Version 2.3.19PR1.0 software (https://www.casaxps.com/) was used for processing XPS data.

### Magnetic measurements

A Vibrating-Sample Magnetometer (VSM) Lakeshore 7300 was used to perform the bulk magnetic measurements. The magnetic measurements of the Py NWs inside de AAO and in the composite at room temperature were made using a sensibility of 10^–2^ emu, acquisition time of 60 min and 13 T of maximum applied field. A magneto-optic Kerr effect (MOKE) magnetometer, NanoMOKE3, was also used to measure a hysteresis curve of the magnetic surface of the composite applying the magnetic field in-plane of the sample between 1.5 and − 1.5 kOe at room temperature.

### Electrochemical characterization

The Electrochemical Impedance Spectroscopy (EIS) study was realized, using an AUTOLAB PGSTAT100, employing a 10 mM [Fe(CN)_6_]^3−^/[Fe(CN)_6_]^4−^ + 0.1 M KNO_3_ aqueous solution. As reference and auxiliary electrode was used Ag/AgCl/Cl^−^(sat) and Graphite bar, respectively. The first and last applied frequency was 10^4^ Hz to 2 × 10^–2^ Hz, respectively, recording 60 frequencies and applying a V_PP_ = 10 mV. The ZView 3.1c software (https://www.scribner.com/programs/zv31c.exe. Copyright 1990-201, Scrib-ner Associates Inc., NC, USA written by D. Johnson) was used for fitting the EIS data.

## Supplementary information

Supplementary Information.
