# Supplementary material for: Permalloy nanowires/graphene oxide composite with enhanced conductive properties
Source: Sci Rep. 2020 Aug 13;10:13742. doi: 10.1038/s41598-020-70512-1 (PMC7426915; doi:10.1038/s41598-020-70512-1)
Supplement: Supplementary file 1 — Supplementary Information. [file 41598_2020_70512_MOESM1_ESM.pdf]

Permalloy nanowires/graphene oxide composite with enhanced conductive properties

Diana M. Arciniegas Jaimes[1], Paulina Márquez[2,3], Alexandra Ovalle[2], Juan Escrig[2,3], Omar Linarez Pérez[4,5,*], and Noelia Bajales[1,6,]**

[1]CONICET, IFEG. Av. Medina Allende s/n, 5000. Córdoba, Argentina.

[2]Universidad de Santiago de Chile. Department of Physics. 9170124. Santiago, Chile.

[3]Center for the Development of Nanoscience and Nanotechnology. 9170124. Santiago, Chile.

[4]Universidad Nacional de Córdoba. Facultad de Ciencias Químicas. Departamento de Fisicoquímica. Haya de la Torre esq. Medina Allende, X5000HUA. Córdoba, Argentina.

[5]CONICET, INFIQC. Haya de la Torre esq. Medina Allende, X5000HUA. Córdoba, Argentina

[6]Universidad Nacional de Córdoba. FAMAF. Medina Allende s/n, Ciudad Universitaria. 5000. Córdoba, Argentina

[*]olinarez@unc.edu.ar

[**]noelia.bajales.luna@unc.edu.ar

The size of monolayer GO from commercial Graphenea which have a flake distribution of sizes of ~1 μm can be seen in **Figure S1**

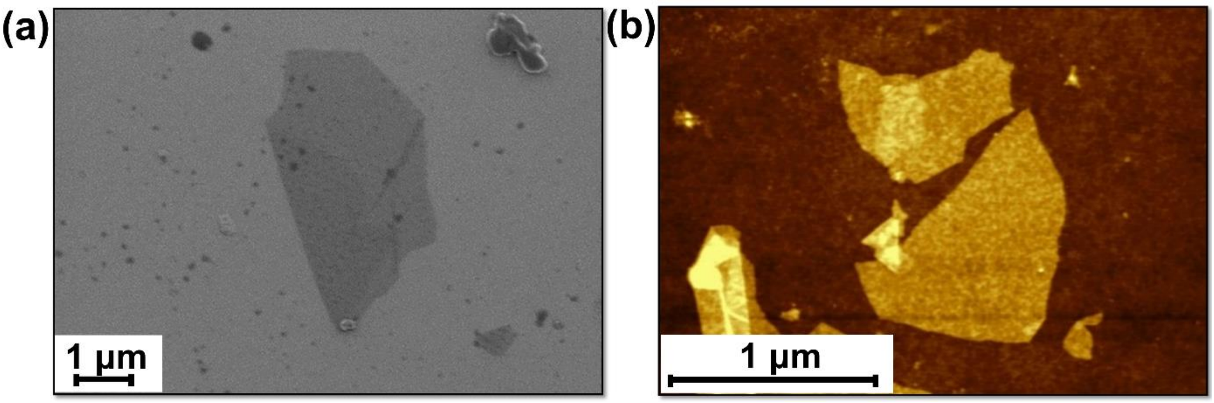

Figure S1. Images of monolayer GO through (a) SEM and (b) AFM.

The cylinder shape model for fitting SAXS data is defined by its radius R and its length L. Its form factor is defined as shown in **Equation 1**:

$$P_{cyl}(q, R, L) = 16 \int_0^1 \left(\frac{J_1\left(qR\sqrt{1-x^2}\right)\sin(qLx/2)}{q^2R\sqrt{1-x^2}Lx} \right)^2 dx \qquad (1)$$

Table S1. Fitting parameters for O1s, C1s, Ni2p and Fe2p XPS spectra for Py NWs/GO and Py NWs samples.

	B.E. (eV)	FWHM (eV)	L/G Mix (%)	Area (%)	B.E. (eV)	FWHM (eV)	L/G Mix (%)	Area (%)
	Py NWs/GO				Py NWs			
O1s								
C-O	532.0	2.6	50	73	531.7	2.2	52	100
Metal-O	530.0	3.5	50	15	---	---	---	---
O-H	527.3	3.1	10	12	---	---	---	---
C1s								
O-C=O/ O-H	288.8	1.5	10	4	289.1	1.8	35	8
C-O-C	286.6	1.8	50	18	285.9	3.5	35	25
Csp^3	284.8	1.8	50	47	284.7	1.5	35	67
Csp^2	283.4	2.9	50	19	---	---	---	---
Not assigned	280.2	3.2	10	12	---	---	---	---
Ni2p								
Ni (II)	855.9	3.5	100	100	856.0	3.5	100	100
Fe2p								
Fe (II)	708.0	2.0	50	50	708.5	1.3	20	30
Fe (II) sat	715.8	5.0	50	24	715.5	6.0	20	33
Fe (III)	710.6	3.3	50	26	710.2	2.7	20	37

The electrochemical parameters obtained from the adjustment with the circuit (I) and (II) of Figure 8c, it is shown in Table S2.

Table S2. Electrochemical parameters obtained from the adjustment.

	R_s (Ω cm_2)	R_{ct} (Ω cm^2)	σ (Ω $cm^{-1}s^{-1/2}$)	Q ($Ω^{-1}$ cm^{-2} s^n)	n	$C_{uncovered}$ ($Ω^{-1}$ cm^{-2} s)	$R_{uncovered}$ (Ω cm^2)
S	7.16 ± 0.02	3.94 ± 0.02	17.75 ± 0.02	(8.4 ± 0.2) $x10^{-5}$	0.8	---	---
GO	15.2 ± 0.2	(54 ± 3) $x10^3$	981.6 ± 37.1	(11.3 ± 0.3) $x10^{-5}$	0.8	(10.1 ± 0.4) $x10^{-5}$	15.4 ± 7.4
Py NWs	10.4 ± 0.1	177 ± 0.8	22.3 ± 0.4	(11.7 ± 0.2) $x10^{-5}$	0.8	---	---
Py NWs/GO	23.3 ± 0.1	158 ± 1.0	229.1 ± 0.8	(5.3 ± 0.1) $x10^{-5}$	0.7	---	---

The length, L, of the Py NWs is ~1 μm as can be seen in **Figure S2**.

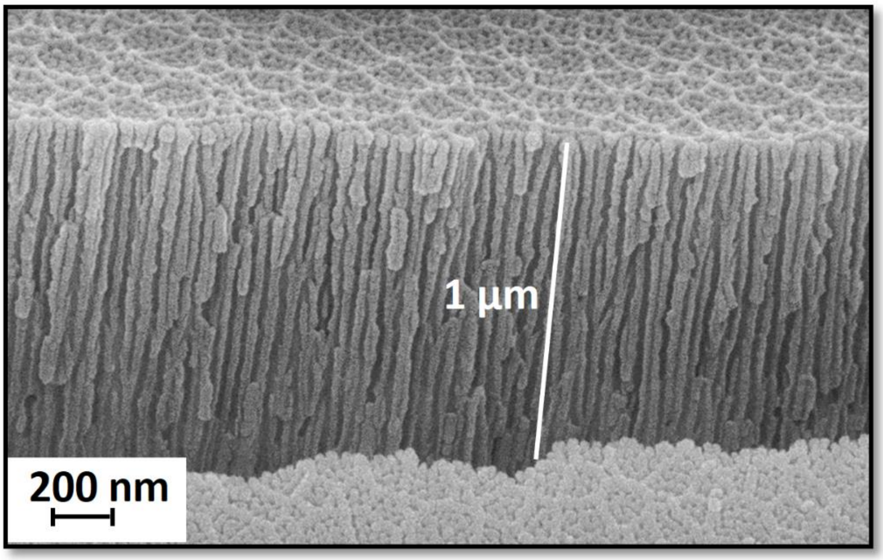

Figure S2. Image of SEM of Py NWs inside AAO template.

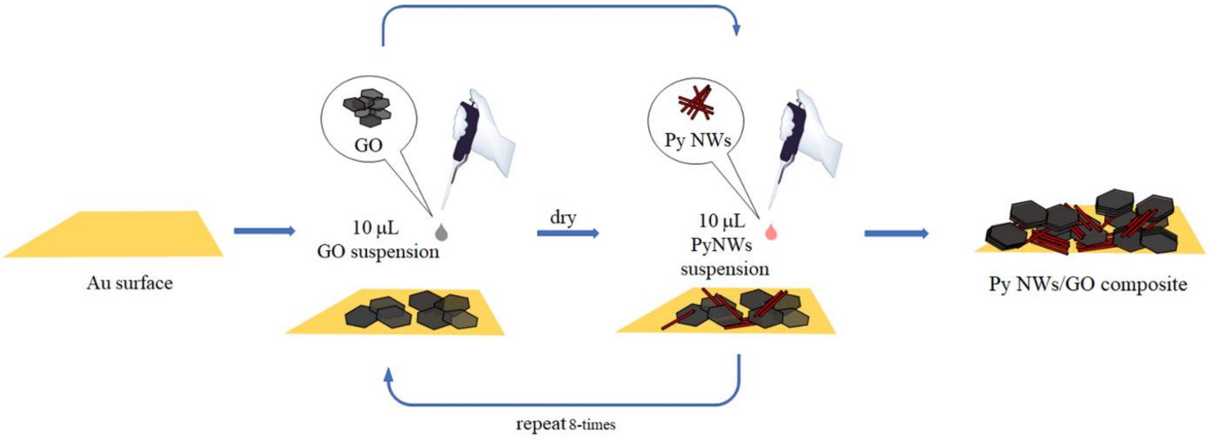

Figure S3. Scheme of the experimental procedure for obtaining the composite.
